# Assessment of the breath alcohol concentration in emergency care patients with different level of consciousness

**DOI:** 10.1186/s13049-014-0082-y

**Published:** 2015-02-06

**Authors:** Annika Kaisdotter Andersson, Josefine Kron, Maaret Castren, Asa Muntlin Athlin, Bertil Hok, Lars Wiklund

**Affiliations:** Hök Instrument AB, Västerås, Sweden; Karolinska Institutet, Department of Clinical Research and Education, Södersjukhuset, Stockholm Sweden; Section of Emergency Medicine, Södersjukhuset, Stockholm Sweden; Department of Medical Sciences, Uppsala University, Uppsala, Sweden; School of Nursing, University of Adelaide, Adelaide, Australia; Department of Emergency Care, Uppsala University Hospital, Uppsala, Sweden; Department of Public Health and Caring Sciences, Uppsala University, Uppsala, Sweden; Department of Surgical Science, Anesthesiology and Intensive Care, Uppsala University, Uppsala, Sweden

**Keywords:** Blood alcohol concentration, Breath alcohol concentration, Patient cooperation, Emergency care patients

## Abstract

**Background:**

Many patients seeking emergency care are under the influence of alcohol, which in many cases implies a differential diagnostic problem. For this reason early objective alcohol screening is of importance not to falsely assign the medical condition to intake of alcohol and thus secure a correct medical assessment.

**Objective:**

At two emergency departments, demonstrate the feasibility of accurate breath alcohol testing in emergency patients with different levels of cooperation.

**Method:**

Assessment of the correlation and ratio between the venous blood alcohol concentration (BAC) and the breath alcohol concentration (BrAC) measured in adult emergency care patients. The BrAC was measured with a breathalyzer prototype based on infrared spectroscopy, which uses the partial pressure of carbon dioxide (pCO_2_) in the exhaled air as a quality indicator.

**Result:**

Eighty-eight patients enrolled (mean 45 years, 53 men, 35 women) performed 201 breath tests in total. For 51% of the patients intoxication from alcohol or tablets was considered to be the main reason for seeking medical care. Twenty-seven percent of the patients were found to have a BAC of <0.04 mg/g. With use of a common conversion factor of 2100:1 between BAC and BrAC an increased agreement with BAC was found when the level of pCO_2_ was used to estimate the end-expiratory BrAC (underestimation of 6%, r = 0.94), as compared to the BrAC measured in the expired breath (underestimation of 26%, r = 0.94). Performance of a forced or a non-forced expiration was not found to have a significant effect (p = 0.09) on the bias between the BAC and the BrAC estimated with use of the level of CO_2_. A variation corresponding to a BAC of 0.3 mg/g was found between two sequential breath tests, which is not considered to be of clinical significance.

**Conclusion:**

With use of the expired pCO_2_ as a quality marker the BrAC can be reliably assessed in emergency care patients regardless of their cooperation, and type and length of the expiration.

## Introduction

Many patients seeking care at the hospital emergency departments (EDs) are under the influence of alcohol, which in many cases implies a differential diagnostic problem [1-3], and assessing the influence from alcohol based on patient anamnesis, clinical signs or characteristics introduce inaccuracies [4,5]. For this reasons early objective alcohol screening is of importance in order not to falsely assign the medical condition to intake of alcohol and thus secure a correct medical assessment [1,3,6,7].

A breathalyzer provides a non-invasive and rapid quantification of the patients’ breath alcohol concentration (BrAC). With use of a conversion factor, called the blood:breath ratio (BBR), the blood alcohol concentration (BAC) can be estimated [8,9]. However, the accuracy of the measured BrAC and thus the estimate of the BAC depend on the duration of the expiration which requires cooperation and good respiratory ability from the person tested [10]. In Sweden most EDs are equipped with breathalyzers but the usability of these devices are limited by the requirement of the patient’s cooperation. For this reason invasive, costly, and time-consuming blood analysis is still widely used.

The objective of this study is to evaluate a breathalyzer prototype which uses expired partial pressure of CO_2_ (pCO_2_) as a quality marker of the breath test. Our hypothesis is that through simultaneous measurement of expired alcohol and the pCO_2_, the BrAC can be reliably assessed regardless of patient cooperation and respiratory ability. The hypothesis is evaluated through comparison of the estimated BrAC and the measured venous BAC.

## Materials and methods

### The study design

#### Study settings and patients

The study was undertaken between November 2010 and June 2011 at two of the largest emergency departments (EDs) in Sweden; Uppsala University Hospital, a level 1 trauma center with approximately 53 000 annual visits, and Södersjukhuset in Stockholm a hospital with nearly 90 000 annual visits. A small number of enrolled nurses working at each ED were assigned to identify and recruit patients over the age of 18 for whom determination of the influence of alcohol would be of clinical benefit, for example patients believed to be sober and patients with variable consciousness.

For each included patient a study protocol was filled in with data regarding age, gender, estimated weight and height, level of consciousness, chief complaint, suspicion and history of alcohol consumption and drug usage. The time for blood alcohol and breath alcohol samplings, and the result of the two analyses were documented. Informed consent was collected in advance from subjects whom were able to be informed or afterwards for the subjects highly under the influence and/or with variable consciousness, at the time of admittance. Data collection from these two EDs was approved by the Regional Ethical Review Board in Uppsala (registration no 2010/048 and 2010/308).

A required study population was predicated from a calculation of the confidence interval (CI) for different samples sizes. At approximately 45 subjects the curve starts to level off and the benefit from including more subjects was therefore minimal. The calculation was based on an assumed bias of 0.068 mg/g and a standard deviation (SD) for difference of 0.0452 mg/g according to a study comparing the BrAC with the BAC [9]. The aim was to recruit 45 patients from each of the two EDs.

## Measurement and procedure

### Measurement of the blood alcohol concentration

At both EDs standard routine involves blood analysis for toxic substances in patients for whom a suspicion of intake of alcohol or other substances exists. Blood sampling and the serum ethanol analysis were performed according to standard procedure and analyzed with gas chromatography at the clinical chemistry department at Södersjukhuset in Stockholm, and with immunoassay analyzer at Uppsala University Hospital.

### Measurement of the breath alcohol concentration

The enrolled nurses were trained to perform breath testing with a handheld breathalyzer prototype with dimensions of 150×85×50 mm, and a weight of less than 200 g. The breath test was initiated by the user from a touch screen PC connected to the breathalyzer, which also presented the result. The breathalyzer utilizes infrared (IR) transmission spectroscopy [11], a highly reliable technique utilized by evidential breathalyzers [12,13]. IR spectroscopy enables continuous and simultaneous measurements of both the expired alcohol and the partial pressure of CO_2_. To ensure low sensitivity to other substances occurring in normal breath a wavelength of 9.5 μm was used for detection of ethanol, whereas a wavelength of 4.3 μm was used for CO_2_.

The breathalyzer continuously sampled 13 seconds of normal breathing through the patient’s mouth and nose, with use of a disposable breathing mask (Ecomask II, size 2, Intersurgical Ltd., U.K), equipped with a bacterial filter (Electrostatic Filter Media MES, Munktell Filter AB, Sweden). The rationale of using CO_2_ for enabling BrAC determination in passive shallow expiration has been subject to previous investigation [14-16]. For estimation of the end-expiratory BrAC (BrACest) the breathalyzer tested uses equation (1) with the assumption that the pCO_2_ in alveolar air is 4.8 kPa with a standard deviation of 10% [17]. From studies of expirograms of CO_2_ recorded from healthy persons and patients with COPD [14-16] a breath sample with a measured pCO_2_ over 1.5 kPa was considered approved. A breath sample with a measured pCO_2_ over 4.8 kPa was considered as a complete expiration and the measured BrAC was considered to be valid as the end-expiratory BrAC. The measurement accuracy of the breathalyzer prototype was ±0.05 mg/l or ±10% of the measured breath alcohol value.

At least two breath tests were performed with each subject. Whether the subject was awake or sleeping/had a lower level of consciousness, and performed a forced or a non-forced expiration was documented. Since the patients were not regarded to have consumed alcohol less than 15 minutes prior to breath testing, no attempt to remove any influence of mouth alcohol was done before testing.

1 $$ \frac{BrA{C}_{est}}{BrA{C}_{meas}}=\frac{pCO{2}_{end- exp}}{pCO{2}_{meas}} $$

*BrAC*_*est*_ *= the breath alcohol concentration estimated to be valid after a forced expiration.*

*BrAC*_*meas*_ *= the breath alcohol concentration measured in the breath sample.*

*pCO2*_*end-exp*_ *= the assumed partial pressure of carbon dioxide after a prolonged, end-expiratory, expiration.*

*pCO2*_*meas*_ *= the measured partial pressure of carbon dioxide measured in the breath sample.*

### Data analysis

The serum ethanol concentration values from the analysis were transformed from mmol/l to mg/g in whole blood, which corresponds to parts per thousand, using the conversion factor recommended for scientific use; 0.0376 [18]. The conversion factor used does not account for any safety margin applied for legal use. Regression analysis and calculation of the Pearson correlation coefficient and the residual standard deviation have been performed. In addition, Bland-Altman analysis [19] and calculation of the mean, upper and lower limits of agreement (LOA) was performed. The LOAs corresponds to a range in which 95% of the differences between two separate measurements of two specimens or tests would be found. For comparison between the blood and breath specimens a blood:breath ratio (BBR) of 2100:1 was used. With consideration to the density of whole blood, a BBR of 2100:1 results in a ratio of 2:1 between the BAC (mg/g) and BrAC, which therefore can be presented as mg/2 l breath [9,18]. The ratio of 2:1 was used for the identity line in the regression analysis plots, and the expression of BrAC in the unit of mg/2 l in the Bland-Altman plots. In addition the BBR for each pair of blood and breath tests was also calculated.

Analyses of one set of paired data per test subject; the blood sample and the first approved breath test (n = 88), and all approved breath tests (n = 201) have been performed. Independent T-tests were performed to analyze the impact on the agreement between the BAC and the BrAC, of whether the subject was passive/active for breath testing and performed a forced/non-forced expiration. A *p*-value of ≤0.05 was chosen as the significant level. All statistical analyses were made using IBM SPSS Statistics version 19.

## Result

### Patient and sample characteristics

Of the 90 patients enrolled two patients were excluded, the first due to methanol poisoning, and the second as no approved breath test (pCO_2_ over 1.5 kPa) was achieved. A total of 88 patients, 35 women (40%) and 53 men (60%), presented to the two EDs were included in the study. Table 1 presents the characteristics of the patients. The mean age of the patients was 45 years (SD ±19, range 18–86). For 35 of the patients (51%) the medical staff considered intoxication from alcohol or tablets to be the main reason for seeking medical care. Twenty-four (27%) of the patients were found to have a BAC of <0.04 mg/g. The mean BAC for the whole population was 1.26 mg/g, whereas the BAC for the alcohol positive patients ranged from 0.15 to 3.46 mg/g, with a mean of 1.73 mg/g. For the patients with a positive BAC (n = 64) the mean measured BrAC was 0.64 mg/l (range 0 to 1.71 mg/l) and the mean BrAC_est_ 0.86 mg/l (range 0 to 2.03 mg/l). This indicates a general upward adjustment of 34% of the BrAC_est_ as compared to the BrAC_meas_. There was also a clear difference in the calculated BBR with respect to use of the BrAC_meas_ and the BrAC_est_, see Table 1 for mean and range of the BBR to be compared with the assumption of a fixed conversion factor, e.g. 2100:1.

**Table 1:** **Patient characteristics and characteristics of the first breath test performed and the blood and breath alcohol measures (n = 88)**

**Data for all patients**	**n**	**Mean**	**SD**	**Range**
Gender: 53 men, 35 women	88			
Age [years]	88	45	±19	18 - 86
BAC [mg/g]	88	1.26	±1.06	0.0 - 3.46
pCO2, first approved breath test [kPa]	88	3.54	±0.88	1.7 - 5.59
No. of negative blood tests [n]	24 (27%)			
No. of test with awake/sleeping subjects [n]	63/15 (not all tests characterized )			
No. of the forced/non-forced breath tests [n]	10/57 (not all tests characterized )			
**Data for the patients with a positive BAC**	**n**	**Mean**	**SD**	**Range**
BAC [mg/g]	64	1.73	±0.85	0.15 - 3.46
Measured BrAC (BrAC_meas_) [mg/l]	64	0.64	±0.36	0.0 - 1.71
Estimated end-expiratory BrAC (BrAC_est_) [mg/l]	64	0.86	±0.43	0.0 - 2.03
Ratio BAC/BrAC_meas_	63	2994	±947	1758 - 7776
Ratio BAC/BrAC_est_	63	2144	±503	1130 - 3632

In total the 88 patients performed 201 breath tests. Of the first approved breath tests performed by each patient, one breath test was found to be false positive with a measured BrAC > 0.10 mg/l (the same applied for the patient’s second breath test) and one breath test was found to be false negative (BAC 0.15 mg/g) (sensitivity 98.4%, specificity 95.8%).

### Relation between the BrAC and BAC

**Figure 1:**
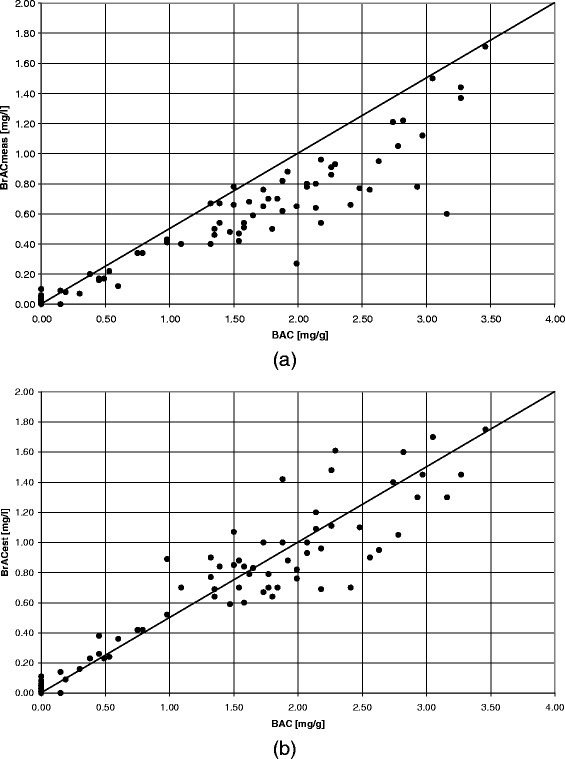
**The correlation between the BAC and the BrAC, where the identity lines represents a BBR of 2100:1. (a)** The BrAC_meas_ gave a clear underestimation of the BAC with 26% (n=88; y=0.368x+0.0092; r=0.94. **(b)** Use of the BrAC_est_ resulted in an underestimation of the BAC of only 6%, (n=88; y=0.466x+0.0465; r=0.94), the reason for this is the reduced effect from difference in cooperation and duration of the expiration performed by the subjects. One clear example of this reduced effect is the two outliers visible in Figure 1a, which are moved into the population in Figure 1b.

In Figure 1a and b (see 1a) the correlation between the BrAC_meas_ and the BAC is presented. The regression equation (y = 0.368x + 0.0092; r = 0.94) indicates that with an assumption of a BBR of 2100:1 (a ratio of 2:1 gives y = 0.5x), the BAC would be underestimated with 26%. If the BrAC_est_ was used to predict the BAC, the underestimation was decreased to 6% (y = 0.466x + 0.046, r = 0.94) (Figure 1a and b (see 1b)). No significant offsets were found for the two measurement series, and the random errors expressed as the residual standard deviations were 0.0147 mg/l and 0.0182 mg/l, respectively.

**Figure 2:**
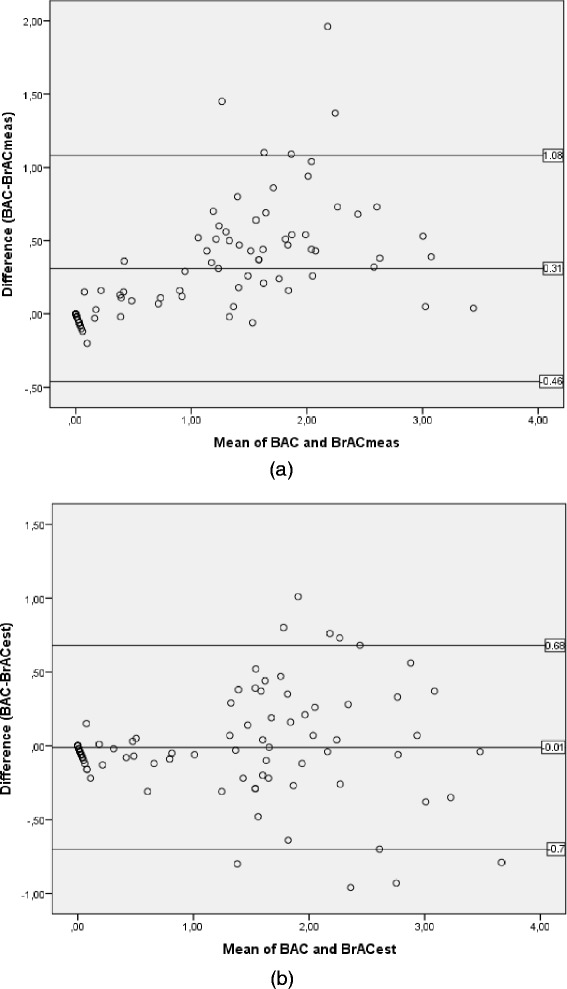
**Analysis of the first breath sample (n=88) illustrating the difference between the BAC and the BrAC with a Bland-Altman plot. (a)** A mean bias of 0.31 mg/g was found between the BAC and the BrAC_meas_, upper limits of agreement (LOA) of 1.09 mg/g and lower LOA of -0.46 mg/g. **(b)** No bias was found between the BAC and the BrAC_est_, upper LOA of 0.68 mg/g and lower LOA of -0.70 mg/g.

With a Bland Altman plot the agreement and differences between the BAC and the BrAC can be illustrated. Analysis of the difference between the BAC and the BrAC_meas_ showed a large positive bias (0.31 mg/g) (Figure 2a and b (see 2a)). Comparing the BAC and the BrAC_est_ showed no bias and a more even distribution (Figure 2a and b (see 2b)). Including paired data for all approved breath tests (n = 201) only indicates a minor change of the mean bias presented in Figure 2a and 2b; from 0.31 mg/g to 0.28 mg/g (upper limits of agreement (LOA) 1.00 mg/g and lower LOA −0.43 mg/g), and from 0.00 mg/g to −0.02 mg/g (upper LOA of 0.68 mg/g and a lower LOA of −0.72 mg/g), respectively.

### Difference in estimated BrAC between the two breath tests

**Figure 3:**
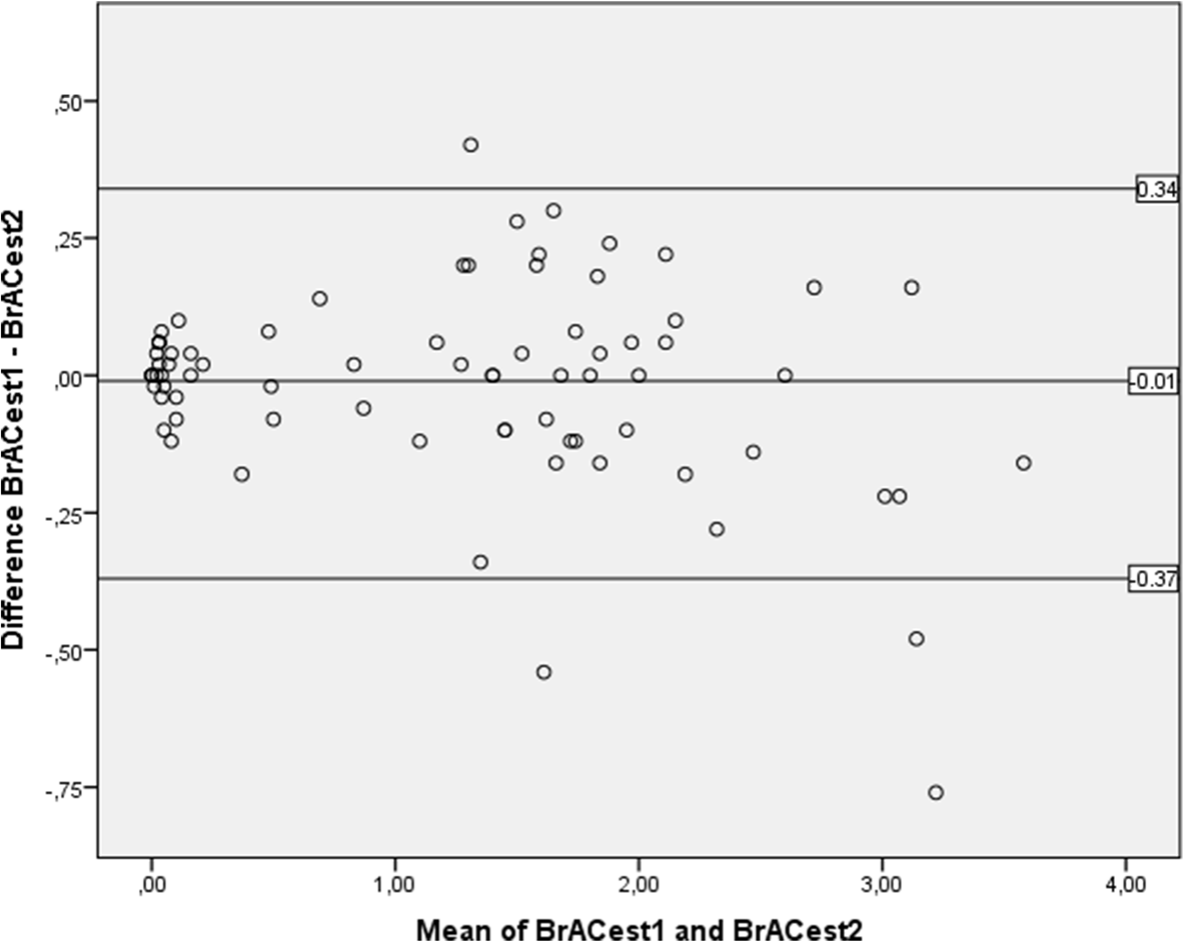
**The Bland-Altman Plot presents the difference in estimated BrAC from two breath tests in relation to the mean of the estimated BrAC for the two breath tests (n = 76).** The plot indicates no bias and an even distribution of the upper and lower LOAs around 0. The LOAs of 0.34 mg/2 l and −0.37 mg/2 l, indicate differences in the BrAC estimated from two sequential breath tests, and reflect the measurement repeatability.

For evaluation of the repeatability of the presented value of the BrAC_est_ a Bland-Altman plot is presented in Figure 3. The data is from two sequential approved breath tests performed by 76 patients. The result indicated no bias, but the upper and lower LOA of 0.34 mg/2 l and −0.37 mg/2 l (equal to mg/g) indicate that there was an evenly distributed difference in BrAC_est_ between the first and second breath test.

### The bias in relation to maximal measured level of pCO_2_

**Figure 4:**
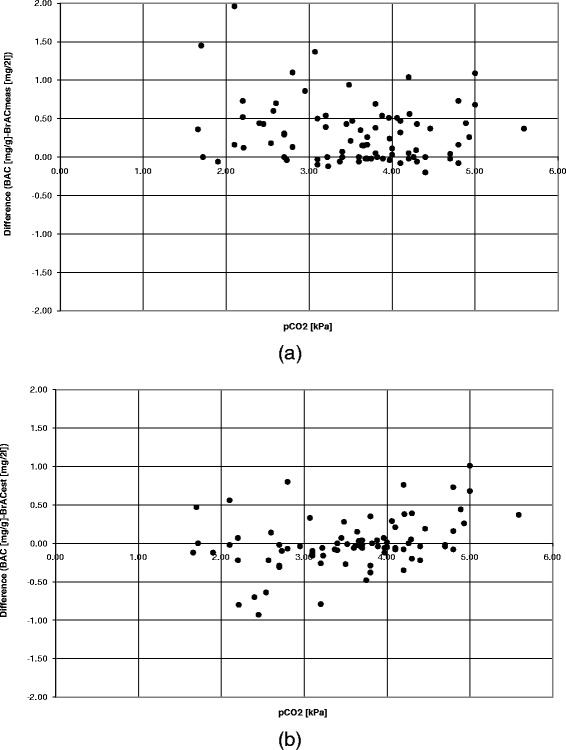
**The bias between the BAC and the BrAC in relation to the pCO**_**2**_ **. (a)** The underestimation of the BrAC_meas_ as compared to the BAC is decreased with increased level of measured pCO_2_, which is achieved with increased length of expiration. **(b)** No bias and a more even distribution around the x-axis indicate a decreased influence from measured pCO_2_ with the use of BrAC_est_.

The bias found between the BAC and the BrAC_meas_ can possibly be reflected in the length of the expiration, and thus the measured level of pCO_2_. For the first approved breath test (n = 88) a mean pCO_2_ of 3.54 kPa was found (range 1.66 – 5.59 kPa). Figure 4a and b (see 4a) presents the bias between the BAC and the BrAC_meas_ in relation to the measured pCO_2_. For the breath tests with a measured pCO_2_ over 4.8 kPa the value of the measured BrAC was used in Figure 4a and b (see 4b). Figure 4a and b (see 4b) illustrates a more even distribution around the x-axis when the difference between the BAC and the BrAC_est_ is plotted in relation to the measured pCO_2_, which indicated less dependence on the pCO_2_, as compared to the results in Figure 4a and b (see 4a).

The influence from the patients’ breath testing performance on the BrAC could also be assessed from the test characteristics documented in the study protocol. A forced or non-forced expiration was not found to have a significant effect (p = 0.09) on the bias between the BAC and the BrAC_est,_ whereas, the bias between the BAC and the BrAC_est_ for awake subjects was significantly (p = 0.02) different from the bias for subjects who slept or had a lower level of consciousness (Table 2).

**Table 2:** **The influence of breath test performance in relation to the mean bias of the BAC and the estimated BrAC (BrAC**_**est**_ **)**

	**N**	**Mean bias between the BAC and the BrAC**_**est**_	**Standard deviation**	**P-value**
**Forced expiration**	10	−0.15	0.39	
**Non-forced expiration**	57	0.05	0.32	0.09
**Awake**	63	−0.03	0.34	
**Sleeping or lowered level of consciousness**	15	0.20	0.37	0.02

A p-value < 0.05 was considered significant.

## Discussion

During the study it was found that the personnel were able to attain approved breath tests from the patient’s normal breathing without problem, after been given minimal user instructions from the test leader or from other enrolled and trained colleagues. The small gentle expiration needed for breath testing is of benefit for the many persons with respiratory impairment, such as asthma and COPD, and variable consciousness who are seeking emergency care.

The results showed that with estimation of the BrAC with use of the expired pCO_2_ as a quality indicator (BrAC_est_), the influence of the patient’s cooperation, passive/forced expirations, or length of expiration is reduced and the measurement accuracy increased. As compared to the reliability of the BrAC measured in the breath (BrAC_meas_) which is clearly related to the level of the patient’s cooperation and length of the expiration (Figures 1a and 2a). This is also illustrated in the more symmetric distribution in (Figure 4b, as compared to 4a).

The results found in this study agree with previous results of ours, with an earlier prototype tested on emergency patients [15]. Another important result is that the variation in BrAC_est_ found between two sequential breath tests (Figure 3) corresponding to a BAC of 0.3 mg/g, is of no clinical significance since a variation of that level would not make any difference in the medical assessment or care of the patient.

Since the BBR depends on many different factors, there is significant controversy regarding the assumption of a fixed BBR in converting between BrAC and BAC. Despite our knowledge of this, a BBR of 2100:1 was used for comparison in Figure 1 and the Bland Altman plots in Figure 2. The actual BBR for a paired data set of a BAC and BrAC is dependent on, for example whether the subject is in the absorption or the elimination phase of alcohol [20], the length of the expiration [10], the attained alcohol concentration [21,22], eventual time elapsed between measurement of the two specimens [8,23], and whether arterial or venous blood are sampled [20]. Concerning the set-up of this study only venous blood samples were analyzed and the recommended maximum time of 30 minutes between the blood test and the breath tests was not exceeded for any of the patients. However, the time elapsed since the patient consumed alcohol was unknown and therefore whether the patient was in the absorption or elimination phase. The effect on the level, and the variability, in BBR as a result of different lengths of expiration can be seen in the BBR calculated for the BrAC_meas_ as compared to the BrAC_est_. With the BrAC_est_, which uses the CO_2_ as a tracer gas in order to reduce the influences from variation in type and duration of the expiration, the mean BBR was significantly reduced (from 2994 to 2144) and the standard deviation of the mean was reduced to half (from 900 to 500).

In addition the patients enrolled in the study showed a large difference in levels of intoxication. In both Figures 1 and 2 it is shown that at BAC below 1 mg/g the distribution is small, whereas it is increased, up to 4 times, at BAC over 1 mg/g. This increase in variability of the BrAC at higher level of BAC could be the result of increased variability in the BBR, which in turn could be related to increased effect from the intoxication level, and if the patient sought emergency care in the absorption or elimination phase of alcohol for which the distribution of alcohol between venous blood and breath are different.

A tendency of increased variability in BBR at increased concentrations has also been found by Sebbane et al. [24]. Sebbane et al. found a BBR of 2615 ± 387 when breath testing ED patients, and concluded that the legal conversion factor of 2000:1 used in France was not appropriate in the ED setting. In Sweden, a BBR of 2100:1 is used in breathalyzers for legal purposes [8]. As compared to 2000:1 and 2100:1, the higher BBR found by Sebbane et al. and for BrAC_meas_ in this study can possibly be explained by the lower grade of cooperation and lower expired volume for patients as compared to healthy subjects, i.e. drunk drivers. However, the results of our study indicate that with use of BrAC_est_ the BBR used for legal purposes in the US and in Sweden (2100:1) is most appropriate for medical applications.

The method of estimating the BrAC with the assumption that the end-expiratory pCO_2_ is 4.8 kPa has been investigated with both healthy subjects and patients with respiratory impairments [14]. When breath testing patients this can introduce inaccuracies, e.g. for patients with respiratory diseases or respiratory distress the BrAC_est_ might be less accurate. In Figures 1b and 2b it is shown that the BrAC_est_ gives a better agreement to a BBR of 2100:1 and an even but somehow increased distribution, as compared to the BrAC_meas_, see Figures 1a and 2a. This increase in variability can indicate that the assumption of a fixed value pCO_2_ value of 4.8 kPa is less appropriate for emergency patients. However, this effect has to be compared to the underestimation of BAC of 26% seen with the BrAC_meas_. Another indication are found in two outliers in BrAC_est_ for breath tests (n = 201) performed on a patient admitted with respiratory distress (n = 2, pCO_2_ = 1.6 kPa and 2.0 kPa, respectively). An additional indication of the inaccuracy that the measurement method introduces by assuming a constant end-expiratory pCO_2_ for all patients is found in Table 2. This shows a significant difference in the mean bias between the BAC and the BrAC_est_, for the breath tests performed on awake patients and those performed on sleeping patients or on patients with a lower level of consciousness.

A problem with the breathalyzer tested which was highlighted during the study was its inability to distinguish between ethanol and methanol in the breath. As compared to breath testing drivers, the sensitivity for methanol is a larger problem in the medical application, since fast and accurate assessment of methanol poisoning is needed because the medical treatment of these patients are completely different to the treatment of ethanol intoxication. However, the IR technology can enable high selectivity [13]. If modified with a second adjacent wavelength for detection of ethanol, selectivity between ethanol and methanol in the breath would be possible with the breathalyzer prototype tested.

Frequent and early objective breath alcohol screening in emergency care could lead to avoidance of differential diagnosis errors, decreased risk of patients being discriminated against or incorrectly treated [5], and decreased costs through decreased use of certain invasive diagnostic procedures [25]. However, state-of-the-art devices available on EDs today have limitations considering usefulness and reliability. This study indicates an innovative solution to these problems. With use of the expired pCO_2_ as a quality marker the breath alcohol concentration can be reliably assessed in emergency care patients regardless of their cooperation.

### Future research

The documented sensitivity of 98.4% and specificity of 95.8% are considered acceptable for this breathalyzer prototype. However, significant technical improvements have been implemented in the next generation, and that within decreased dimensions. The latest technology provides opportunities for continued research of the application in regular clinical use. Areas of particular interests are diagnostic efficiency and the impact on healthcare economics. Additionally, more research is required to investigate the practical usability of this kind of handheld breathalyzer to make it as user-friendly as possible in the ED context.
